# Influence of the Residual Gensini Score on Prognosis of Patients With ST‐Elevation Myocardial Infarction

**DOI:** 10.1002/ccd.70633

**Published:** 2026-04-21

**Authors:** Batric Popovic, Nassima Djaballah, Mirela Tomic, Renaud Fay, Victorine Fraichot, Florian Eggenspieler, Pierre Adrien Metzdorf, Jeanne Varlot, Edoardo Camenzind

**Affiliations:** ^1^ CHRU‐Nancy, Department of Cardiology Université de Lorraine Nancy France

## Abstract

**Background:**

The residual Gensini score (rGS) was developed to quantify the severity of coronary atheroma burden after coronary revascularisation. The predictive value of the rGS for clinical outcomes in patients with ST‐segment elevation myocardial infarction (STEMI) remains unexplored.

**Methods:**

Our retrospective study included 1034 consecutive patients who presented with STEMI between 2016 and 2020. Patients were stratified based on the third tertile of rGS values: rGS ≤ 16 (Group 1) and rGS > 16 (Group 2).

**Results:**

Compared with patients in group 1, epicardial and microvascular perfusion were significantly impaired in group 2 as evidenced by poorer final flow grade TIMI 3 (89% vs. 74%, *p* < 0.0001) and lower complete ST resolution (STR) rates > 70% (56% vs. 49%, *p* = 0.007). In the unadjusted analysis, excess mortality in group 2 was observed early (45‐day mortality rate: 6% vs. 12%, *p* = 0.0005) and persisted during the 2‐year follow‐up (9% vs. 18%, *p* < 0.0001). After stabilized Inverse Exposure Probability Weighting (sIEPW) adjustment, the early mortality of the patients was similar in both groups: 45‐day mortality: 10% vs. 9%, *p* = 0.45, Harrell's c‐index 40%. However, the rGS remained associated with worse prognosis thereafter: 1 year mortality: 11% versuss 21%, Harrell's c‐index: 61%, *p* < 0.0001.

**Conclusion:**

In a real‐world cohort of patients with STEMI, rGS is associated with a worse long‐term prognosis as from 1 year follow‐up, but without significantly stratifying early follow‐up. These findings provide important insight with regard to the optimal use of angiography scoring system as prognostic factor. (ClinicalTrials.gov Identifier: NCT05679843).

## 1 Introduction

Despite advances in secondary preventive strategies, myocardial infarction (MI) remains associated with a substantial risk of recurrent cardiovascular events [1, 2]. This underscores the importance of prognostic stratification in this population [3, 4, 5].

Various angiographic scoring systems, such as the Syntax score [6, 7] or the Gensini score [8, 9, 10], have been developed to assess the severity of coronary artery disease (CAD) by evaluating parameters related to individual coronary artery lesions. These scoring systems have also been tested for their ability to predict morbidity and mortality following the index event. However, these scores primarily quantify either the overall atherosclerotic burden of the coronary tree or the atherothrombotic burden corresponding to the culprit lesion. More recently, the residual GS (rGS) as assessed post‐percutaneous coronary intervention (PCI) has been explored in various clinical settings [11]. However, evidence regarding its utility in patients following STEMI remains limited. This study aimed to evaluate the impact of coronary atheroma burden, as determined by the rGS, on short‐ and long‐term all‐cause mortality in patients with STEMI.

## 2 Materials and Methods

### 2.1 Study Population

A single‐centre, retrospective, consecutive, observational cohort study was conducted, including patients presenting with STEMI and treated with primary PCI (pPCI) at the University Hospital of Nancy between 2016 and 2020.

STEMI was defined as follows: (1) continuous chest pain lasting more than 20 min; (2) ST‐segment elevation ≥ 1 mm in ≥ 2 contiguous leads on a 12‐lead electrocardiogram or left bundle branch block; and (3) treatment within 24 h of pain onset.

The study protocol has been approved by our Institution's ethics committee, with written consent obtained from the patients, and the study protocol conforms to the ethical guidelines of the Declaration of Helsinki.

### 2.2 Procedural Characteristics

During the prehospital phase, patients received as premedication acetylsalicylic acid and non‐fractionated heparin intravenously, and a P2Y12 inhibitor orally (ticagrelor 180 mg, prasugrel 60 mg, or clopidogrel 600 mg as a loading dose) as early as possible. Glycoprotein IIb/IIIa inhibitors were administered during the revascularisation procedure at the physician's discretion. All patients underwent invasive coronary angiography and transthoracic echocardiography. Additional imaging modalities (transoesophageal echocardiography, optical coherence tomography, magnetic resonance imaging, or computed tomography) were performed according to the treating physician's discretion.

### 2.3 Definitions

#### 2.3.1 Residual Gensini Score (rGS)

The atheroma burden was quantified using a recognised angiographic scoring system developed by Gensini et al. [8] Two experienced operators reviewed all coronary angiograms (CAG). At least two orthogonal angiographic projections were used to assess the culprit vessels and lesions. Each coronary artery stenosis was assigned a severity score based on its location and degree. Stenoses of 1%–25%, 26%–50%, 51%–75%, 76%–90%, 91%–99%, and total occlusion were scored as 1, 2, 4, 8, 16, and 32, respectively. These scores were then adjusted by a factor depending on their location in the coronary tree: 5 for the left main coronary artery, 2.5 for the proximal segment of the left anterior descending (LAD) coronary artery and circumflex artery, 1.5 for the mid‐segment of the LAD coronary artery, 1 for the distal segment of the LAD coronary artery, right coronary artery, posterior descending artery, and obtuse marginal artery, and 0.5 for other segments. The Gensini Score was computed by summing the scores of all coronary arteries. The rGS was calculated using the last frame of the initial PCI angiogram or the planned PCI angiogram if a staged revascularisation of non‐culprit lesions was planned during the index hospitalisation [11].

Non‐culprit lesion revascularization, when indicated, was performed either during the index hospitalization or, in all cases, within 3 weeks following the initial MI. For patients who died during the index hospitalization before any staged revascularization could be undertaken, the rGS was calculated using the last frames of the initial culprit‐vessel PCI angiogram.

#### 2.3.2 Reperfusion Analysis

Electrocardiography (ECG) was performed during the prehospital phase (preprocedural ECG), and within 1 h following pPCI (postprocedural ECG). ST‐segment elevation was measured in the lead showing the highest value. ST‐segment elevation resolution (STR) post‐pPCI was calculated as a percentage. Complete STR was defined as a reduction of at least 70% in ST‐segment elevation.

#### 2.3.3 Angiography

Two different angiographic incidences were used during PCI to assess the TIMI flow grade, ranging from 0 to 3. TIMI 0 was defined as no perfusion, TIMI 1 as penetration without perfusion, TIMI 2 as partial reperfusion, and TIMI 3 as complete perfusion. Distal embolisation was defined as an abrupt “cut‐off” in the coronary artery blood flow of the culprit artery or one of its branches downstream of the angioplasty site; this was not included in the TIMI flow‐grade calculations.

#### 2.3.4 Cardiogenic Shock

Cardiogenic shock was defined as sustained systolic arterial pressure below 90 mmHg that was unresponsive to fluids and required inotropic support.

### 2.4 Clinical Events

Clinical outcomes included the 45‐day and 1‐year all‐cause mortality and/or recurrent acute coronary syndrome at 1 year. Further all‐cause mortality up to 2‐year follow‐up was obtained via direct telephone contact of the patient's general physician to complete a comprehensive standardised questionnaire. In case of death, the patient's physician or hospital records were consulted to determine the dates of death and/or possible intercurrent events (e.g., acute coronary syndrome).

### 2.5 Statistical Analysis

All analyses were carried out using SAS R9.8 (SAS Institute, Cary, NC). The overall 2‐tailed significance level was set to *p* < 0.05. Continuous variables were described as the median (1st and 3rd quartiles), whereas the categorical factors have been presented as the frequency (proportion).

Comparisons among the 2 groups were carried out using the Mann−Withney's and Chi‐Square tests (or Fisher's exact test when appropriate) for continuous and categorical variables, respectively.

The comparison of event rates in each group was performed using the Log‐rank test and illustrated by the corresponding Kaplan–Meier survival curves. The independent effects of rGS on the event rates were estimated before and after adjustment for potential confounders among the baseline characteristics using stabilized Inverse Exposure Probability Weighting (sIEPW) [12]. Event rates were estimated as the mortality rates at the 45‐day, 1‐year, 1‐year mortality or recurrent acute coronary syndrome, and 2‐year mortality follow‐up.

## 3 Results

### 3.1 Population Characteristics

Overall, 1034 consecutive patients with STEMI who underwent pPCI were included in the analysis. Patients were stratified based on the third tertile of rGS values into two groups rGS ≤ 16 (group 1) and rGS > 16 (group 2). The baseline characteristics and initial clinical presentation of the two groups are summarised in Table 1.

**Table 1:** Baseline characteristics of the population according to rGS.

Variables		Group 1: Patients with rGS ≤ 16		Group 2: Patients with rGS > 16	
	*n*	Q2 (Q1–Q3)^a^ or n (%)	*n*	Q2 (Q1–Q3) or n (%)	*p*
Demographic characteristics					
Age (years)	689	59 (50–69)	345	67 (55–77)	< 0.0001
Female sex, *n* (%)	689	499 (72%)	345	254 (74%)	0.68
Body mass index (kg/m^2^)	632	26.7 (24.2−29.6)	306	26.7 (24.2−29.4)	0.91
Cardiovascular risk factors, *n* (%)					
Diabetes mellitus	689	91 (13%)	345	75 (22%)	0.0004
Hypertension	689	298 (43%)	345	191 (55%)	0.0002
Hyperlipidemia	689	309 (45%)	345	183 (53%)	0.013
Smoker (past or current)	689	355 (52%)	345	131 (38%)	< 0.0001
Comorbidities, *n* (%)					
History of myocardial infarction	689	45 (7%)	345	29 (8%)	0.27
History of coronary revascularization	2252	57 (8%)	345	44 (13%)	0.003
History of stroke	689	16 (2%)	345	10 (3%)	0.58
Peripheral artery disease	689	11 (2%)	345	22 (6%)	< 0.0001
Chronic obstructive pulmonary disease	689	33 (5%)	345	11 (3%)	0.23
Inflammatory disease	689	19 (3%)	345	10 (3%)	0.90
Previous or current neoplasia	689	18 (3%)	345	13 (4%)	0.30
In‐hospital assessment					
Delay 1st symptoms‐balloon (h), Q2 (Q1–Q3)	666	3.0 (2.0−6.0)	333	4.0 (2.0−7.0)	0.007
Prehospital cardiac arrest, *n* (%)	689	97 (14%)	345	61 (18%)	0.13
Cardiogenic shock, *n* (%)	689	64 (9%)	345	56 (16%)	0.001
Acute heart failure without cardiogenic shock, *n* (%)	689	60 (9%)	345	40 (12%)	0.14
Renal insufficiency, *n* (%)	689	79 (11%)	345	62 (18%)	0.004
GRACE risk score, *n* (%)	689		345		
< 113		451 (65%)		188 (54%)	< 0.0001
113–133		24 (3%)		3 (1%)	
> 133		214 (31%)		154 (45%)	
Atrial fibrillation, *n* (%)	689	54 (8%)	345	47 (14%)	0.003

Abbreviation: GRACE, Global Registry of Acute Coronary Events.

^a^ Q2 (Q1−Q3): Median (1st−3rd quartiles).

Compared with patients in group 1, those in group 2 were older (59 vs. 67 years, *p* < 0.0001) and had a greater prevalence of cardiovascular risk factors, including diabetes mellitus (13% vs. 22%, *p* < 0.0004), hypertension (43% vs. 55%, *p* < 0.0002), and hyperlipidaemia (45% vs. 53%, *p* = 0.013). Conversely, smoking was more frequent in group 1 (52% vs. 38%, *p* = 0.0001). Furthermore, comorbidities such as peripheral artery disease (2% vs. 6%, *p* < 0.0001) and atrial fibrillation (8% vs. 14%, *p* = 0.003) were more prevalent in group 2. The median delay between the onset of symptoms and balloon placement was significantly longer in Group 2 (3 vs. 4 h, *p* = 0.007). Cardiogenic shock was observed in 12% of the patients and was significantly more common in group 2 (9% vs. 16%, *p* = 0.001). Pre‐hospital cardiac arrest occurred in 158 patients (15%) and tended to be more frequent in group 1 (14% vs. 18%, *p* = 0.13).

Table 2 presents the procedural characteristics. The location of the culprit lesion differs significantly (*p* < 0.007) between the 2 groups, with a markedly lower number of LAD in Group 2 (42% vs. 34%). The culprit lesion was proximal in the majority of cases (87% vs. 92%, *p* = 0.007). Epicardial and microvascular perfusion, as indicated by a lower post‐PCI TIMI flow grade 3 (89% vs. 74%) and reduced complete STR ≥ 70% rate (56% vs. 49%), also differed in both groups (*p* < 0.01). No significant differences were observed in troponin or creatine phosphokinase levels between the groups. Similarly, the median LVEF was 50% across the entire population, with no significant differences between the groups.

**Table 2:** Angiographic findings and postprocedural results in the population according to rGS.

Variables	*N*	Group 1: Patients with rGS ≤ 16	*N*	Group 2: Patients with rGS > 16	*p*
		Q2 (Q1–Q3) or *n* (%)		Q2 (Q1–Q3) or *n* (%)	
Angiography					
Location of the culprit lesion, *n* (%)					
Left anterior descending artery	689	290 (42%)	345	117 (34%)	
Left circumflex artery		292 (42%)		152 (44%)	
Right coronary artery		89 (13%)		55 (16%)	0.007
Left main coronary artery		4 (1%)		13 (4%)	
Diagonal artery		14 (2%)		8 (2%)	
Stent thrombosis	689	25 (4%)	345	19 (6%)	0.16
Proximal coronary occlusion	689	598 (87%)	345	319 (92%)	0.007
Pre‐PCI flow grade					
TIMI 0–1	689	456 (66%)	345	242 (70%)	
TIMI 2		69 (10%)		35 (10%)	0.32
TIMI 3		164 (24%)		68 (20%)	
Biological data					
Creatine phosphokinase (UI/L)	678	1629 (743−3423)	336	1546 (826−2967)	0.43
Standard troponin I (µg/L)	367	51.6 (19.9−80.0)	165	48.9 (18.6−80.0)	0.87
High‐sensitivity troponin (ng/L)	320	47 (18−119)	176	47 (20−102)	0.99
Reperfusion strategies used, *n* (%)					
Primary angioplasty (stent or balloon)	689	662 (96%)	345	327 (95%)	0.33
Stent implantation	662	597 (90%)	327	253 (77%)	<0.0001
Direct stenting	485	351 (72%)	211	142 (67%)	0.18
Delayed stenting	689	15 (2%)	345	10 (3%)	0.48
Manual thrombectomy	427	74 (17%)	252	37 (15%)	0.37
GIIb/IIIa inhibitors use	659	128 (19%)	338	64 (19%)	0.85
Reperfusion results					
Post‐PCI flow grade					
TIMI 0–1	689	10 (1%)	345	36 (10%)	
TIMI 2		69 (10%)		53 (15%)	<0.0001
TIMI 3		610 (89%)		256 (74%)	
Post‐PCI distal embolization	614	135 (22%)	261	67 (26%)	0.24
LVEF (%),	689	50 (40–55)	345	50 (40–55)	0.18
<35%, *n* (%)	689	57 (8%)	345	42 (12%)	
35%−45%, *n* (%)		228 (33%)		112 (32%)	0.13
>45%, *n* (%)		404 (59%)		191 (55%)	
ST‐segment resolution on ECG					
Mean ST‐segment resolution:	623	75 (50−100)	316	67 (33−100)	0.0022
Complete > 70%, *n* (%)	623	346 (56%)	316	156 (49%)	
Partial: ≥ 30 and < 70%, *n* (%)		181 (29%)		85 (27%)	0.007
None: < 30%, *n* (%)		96 (15%)		75 (24%)	

Abbreviations: LVEF, left ventricular ejection fraction; PCI, percutaneous coronary intervention; Q2 (Q1−Q3), Median (1st−3rd quartiles); TIMI, thrombolysis in myocardial infarction.

Discharge treatments were comparable between the groups, with consistent prescription rates for antithrombotic agents, statins, beta‐blockers, and ACEIs/ARBs.

### 3.2 Survival Analysis

**Figure 1:**
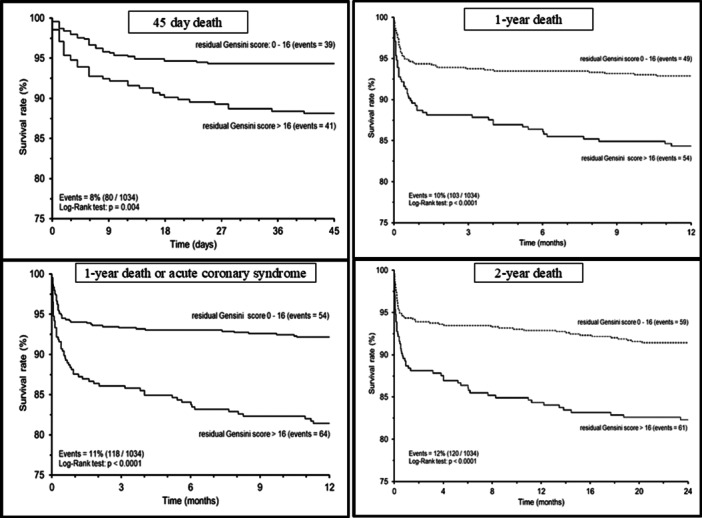
The 45‐day all‐cause mortality, recurrent acute coronary syndrome recorded at 1 year, and the 2‐year all‐cause mortality: full *y*‐axis Kaplan–Meier estimated survival before adjustment in the Group 1 and Group 2.

**Figure 2:**
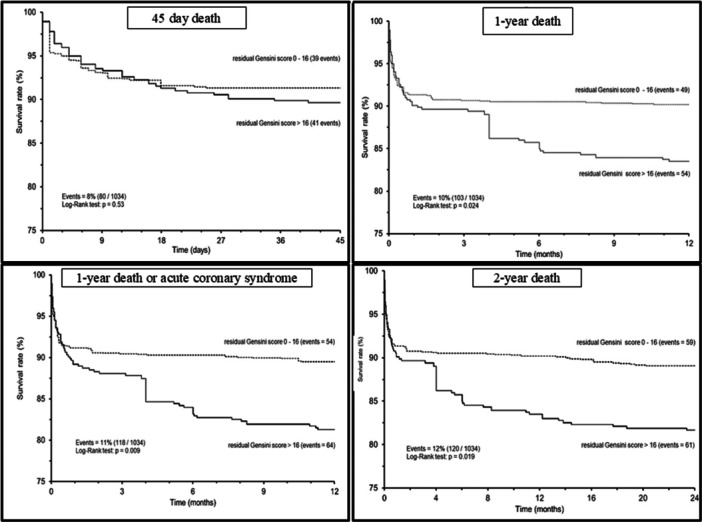
The 45‐day all‐cause mortality, recurrent acute coronary syndrome recorded at 1 year, and the 2‐year all‐cause mortality: full *y*‐axis Kaplan–Meier estimated survival after sIEPW adjustment in the Group 1 and Group 2.

All patients were followed for 2 years after discharge, as illustrated by Kaplan–Meier survival curves in Figures 1 and 2. The unadjusted analysis revealed consistently higher mortality in Group 2, with excess mortality occurring early (45‐day mortality rate: 6% vs. 12%, *p* = 0.0005) and confirmed up to 2‐year follow‐up (9% vs. 18%, *p* < 0.0001) (Figure 2).

After sIEPW adjustment, the early prognosis of the patients was similar in both groups: 45‐day mortality: 10% vs 9%, (Harrell's c‐index 40% *p* = 0.45). However, the rGS remained associated with a worse prognosis thereafter: 1 year mortality: 11% vs 21%, (Harrell's c‐index: 61%, *p* < 0.0001) and 2‐year mortality 12% vs 22% (Harrell's c‐index 60%, *p* < 0.0001).

## 4 Discussion

Although rGS is positively correlated with post‐procedural MI in patients undergoing elective coronary artery interventions [11], evidence linking rGS with mortality in patients with STEMI is limited. To our knowledge, this study is the first to report the association between rGS and mortality in patients with STEMI treated with pPCI. Three major findings were identified:

1. Patients with rGS > 16 were older and had a higher prevalence of cardiovascular risk factors.
2. Early excess mortality was observed in patients with rGS > 16 compared to those with rGS ≤ 16, as confirmed during the 2‐year follow‐up in the unadjusted analysis.
3. After sIEPW adjustment, the early prognosis of patients was similar in both groups at 45 day follow up, whereas the rGS remained associated with a worse prognosis thereafter.

Patients with acute STEMI are at high risk of both short‐ and long‐term morbidity and mortality [13], making risk stratification essential. While validated patient‐based risk scores are widely used to predict outcomes in STEMI [13], angiographic data from pPCI remain a critical indicator of global coronary atherosclerotic burden but are frequently relied upon by practitioners as surrogate prognostic factors. Various angiographic scoring systems have been associated with early and long‐term outcomes in patients with STEMI treated with pPCI. The Syntax Score [6, 7] was developed to objectively evaluate the anatomical complexity of stenotic lesions in patients with multi‐vessel disease. The Syntax Score has been applied in multiple clinical contexts, including STEMI, where a higher score is linked to mortality and adverse cardiovascular events [14]. However, the Syntax Score provides only a partial representation of the coronary atherosclerotic burden. It distinguishes between occlusive (100%) and non‐occlusive (50%–99%) lesions, categorising stenosis as severe if it reduces the luminal diameter by ≥ 50%, and considers only vessels ≥ 1.5 mm. The Gensini score [8], in contrast, accounts for lesions causing < 50% stenosis and those affecting vessels with a luminal diameter < 1.5 mm. This score has been associated with patient prognosis after adjustment for confounding factors. Since the introduction of the Syntax and Gensini scoring systems, additional post‐pPCI scores have been developed as indicators of revascularization outcomes [11, 15, 16].

The rGS quantifies the remaining atheroma burden after revascularization, whereas the Gensini score reflects both the residual atheroma and the thrombotic burden of the culprit lesion. In our study, after appropriate adjustment, residual atheroma following PCI was not associated with increased early mortality but appeared to be primarily linked to mortality from 1 year follow‐up onwards. Indeed, relying solely on an anatomical score to assess such a complex pathophysiological process may be insufficient for accurately predicting both short‐term and long‐term outcomes following myocardial infarct. The most intuitive explanation is the predominant role of clinical features in determining early outcomes, which often outweighs the influence of the extent of CAD.

Indeed, early prognosis after MI is primarily driven by the in‐hospital course, including recurrent ischemic events, arrhythmias, and, more commonly, hemodynamic instability. Patients who experience adverse events during this period are thought to exhibit a persistent proinflammatory state following the acute episode, which predisposes them to subsequent complications [17, 18]. During the long‐term phase, the rGS may complement established clinical risk models to improve post‐STEMI risk stratification. In this context, a high rGS could help identify patients who may benefit from intensified secondary prevention strategies as well as closer clinical surveillance for recurrent ischemic events or heart failure progression.

From a mechanistic perspective, a higher rGS was associated with impaired epicardial and microvascular reperfusion, as indicated by lower TIMI flow grade 3 and reduced complete ST‐segment resolution. This finding suggests that a diffuse residual atherosclerotic burden may be associated with increased distal embolization, microvascular dysfunction, and a persistent pro‐inflammatory state, thereby contributing to greater myocardial injury and increased vulnerability to late adverse events, including recurrent ischemia, heart failure, and arrhythmias.

Different risk scores recommended by ESC guidelines have been developed for early risk assessment after MI [13]. These models have been formulated into clinical risk scores, including the extent of myocardial damage, the achievement of successful reperfusion, and the presence of clinical markers, especially older age, tachycardia, hypotension, Killip class > I, anterior MI, renal function, history of heart failure, peripheral arterial disease, or anaemia [5, 19].

However, in our study, the coronary atheroma burden remained a strong prognostic factor for adverse events in patients with STEMI, after 1 year follow‐up—and possibly even earlier. For the first time, this study showed that the rGS, an angiographic scoring system, has relevant prognostic value for predicting mortality after MI. While clinical scoring systems are likely more appropriate for assessing very early prognosis after STEMI, the current angiographic score remains relevant for long‐term risk stratification, in contrast to the Syntax score, which had been designed more to evaluate acute procedural risk. The current data on global coronary plaque‐burden assessment confirm the importance of an aggressive post‐infarction management of plaque–stabilization and potentially regression program.

## 5 Limitations

First, the observational nature and the retrospective, single‐centre design, as well as the limited number of patients, may have affected the generalisability of these findings. A sensitivity analysis separately addressing mortality and non‐fatal ischemic events could not be meaningfully performed due to the very limited number of recurrent ischemic events observed during the follow‐up.

Moreover, the absence of systematic lesion‐level ischemia assessment, physiological guidance, or intravascular imaging for plaque characterization and stent optimization, together with the lack of longitudinal data on adherence to secondary prevention and risk‐factor control during follow‐up, may have influenced our results.

## 6 Conclusion

In a real‐world cohort of MI patients treated with pPCI, residual Gensini angiographic score—a measurement of global coronary atherosclerotic burden—is associated with a higher long‐term mortality. These findings suggest the utility of this score system as surrogated indicator of long‐term outcome and underscore the importance of an aggressive secondary prevention.

## Funding

The authors have nothing to report.

## Conflicts of Interest

The authors declare no conflicts of interest.

## Supporting information

Supporting File

## Data Availability

The data that support the findings of this study are available from the corresponding author upon reasonable request.
