# Supplementary material for: Influence of the Residual Gensini Score on Prognosis of Patients With ST‐Elevation Myocardial Infarction
Source: Catheter Cardiovasc Interv. 2026 Apr 21;108(1):76–83. doi: 10.1002/ccd.70633 (PMC13331591; doi:10.1002/ccd.70633)
Supplement: Supplementary file 1 — Supporting File [file CCD-108-76-s001.docx]

**Supplemental data:**

**Statistical details for sIEPW calculation**

Thirty-one potentially confounding factors were identified among baseline characteristics of patients with complete observations, significant or not, correlated or not (non parcimonious model):

**Demography**: age and male gender, **Cardiovascular risk factors**: diabetes mellitus, hypertension, dyslipidemia, smoker (past or current), Grace score (class). **Comorbidities**: previous myocardial infarction, previous coronary revascularization, previous stroke or transient ischemic attack, chronic obstructive pulmonary disease, previous/current neoplasia, inflammatory disease,. **Admission - In-hospital assessment**: acute heart failure, acute renal failure, total number of cardiogenic shocks (pre- or in - hospital), pre-hospital cardiogenic shock, pre-hospital cardiac arrest, right ventricular MI, IACPB / ECMO, atrial fibrillation. **Angiographic characteristics**: number of affected coronary arteries (class), culprit coronary lesion, LVEF (class), stent thrombosis, proximal coronary disease, embolic disease, ectasia, TIMI score at admission (before percutaneous coronary intervention, flow grade 0-1, 2, 3) . R**eperfusion strategies**: urgent target vessel angioplasty, delayed stenting.

**Statistical method: standardized Inverse Exposure Probability Weighting**

Reference 12: Brookhart MA, Wyss R, Layton JB, Sturmer T. Propensity score methods for confounding control in nonexperimental research. Circ Cardiovasc Qual Outcomes 2013;6:604-11.

For each patient i in each clinical group j, a propensity score (PSij) was computed as the probability of exposure to risk (CE Yes vs No) at admission given their set of potentially confounding factors using multinomial logistic regression. Thus, PSij can be seen as a surrogate of the patient profile of confounders at admission. The Inverse Exposure Probabilty Weighting (IEPWij) was derived as 1/PSij. Finally, the stabilized IEPWij (sIEPWij) was computed as the product of IEPWij by the marginal frequency of the group j in the whole sample (nj / N). Eventually sIEPWij was used in a multinomial logistic regression model with mortality as event, risk exposure as main explicative variable and sIEPWij as a weighting factor representing the individual baseline profile of each patient. Then, combining all baseline potential confounders in an unique score, the estimation of the relation between exposure and outcome reduces to a single 2-factor model: *outcome (event) = risk exposure (causal factor) and individual profile (adjustment factor)*.
